# New Targets for* Zika* Virus Determined by Human-Viral Interactomic: A Bioinformatics Approach

**DOI:** 10.1155/2017/1734151

**Published:** 2017-12-12

**Authors:** Eduardo Esteves, Nuno Rosa, Maria José Correia, Joel P. Arrais, Marlene Barros

**Affiliations:** ^1^Universidade Católica Portuguesa, Center for Interdisciplinary Research in Health (CIIS), Institute of Health Sciences (ICS), Viseu, Portugal; ^2^Department of Informatics Engineering (DEI), Centre for Informatics and Systems of the University of Coimbra (CISUC), University of Coimbra, Coimbra, Portugal

## Abstract

Identifying ZIKV factors interfering with human host pathways represents a major challenge in understanding ZIKV tropism and pathogenesis. The integration of proteomic, gene expression and Protein-Protein Interactions (PPIs) established between ZIKV and human host proteins predicted by the OralInt algorithm identified 1898 interactions with medium or high score (≥0.7). Targets implicated in vesicular traffic and docking were identified. New receptors involved in endocytosis pathways as ZIKV entry targets, using both clathrin-dependent (17 receptors) and independent (10 receptors) pathways, are described. New targets used by the ZIKV to undermine the host's antiviral immune response are proposed based on predicted interactions established between the virus and host cell receptors and/or proteins with an effector or signaling role in the immune response such as IFN receptors and TLR. Complement and cytokines are proposed as extracellular potential interacting partners of the secreted form of NS1 ZIKV protein. Altogether, in this article, 18 new human targets for structural and nonstructural ZIKV proteins are proposed. These results are of great relevance for the understanding of viral pathogenesis and consequently the development of preventive (vaccines) and therapeutic targets for ZIKV infection management.

## 1. Introduction

The Flaviviridae family,* Flavivirus* genus, consists of a variety of viruses transmitted by blood-feeding arthropod species, several of which represent emergent or reemergent pathogens including* Zika* (ZIKV),* Dengue* (DENV),* Yellow Fever* (YFV),* Japanese Encephalitis* (JEV), and* West Nile* (WNV) viruses. ZIKV, a previously neglected member of the genus, has recently been the subject of concern and research since it has been linked to congenital developmental deficits and neurological syndromes [1–8].

*Flavivirus* virions are composed of a single positive-strand RNA genome, packaged by the viral capsid protein (C) in a host-derived lipid bilayer and surrounded by 180 copies of two structural proteins, envelope (E) and membrane (M) [9, 10]. The genome is translated into a single polyprotein and subsequently cleaved by viral and host proteases into three structural proteins (C, prM/M, and E) and seven nonstructural (NS) proteins (NS1, NS2A, NS2B, NS3, NS4A, NS4B, and NS5) [9, 11].

A successful innate immune response by the host depends on the efficient detection of the invading pathogen.* Flavivirus* use their structural glycoproteins to attach to the host cell, interacting with several receptors, which trigger endocytosis pathways. One of the proteins important in this process is structural E protein with plays a role in receptor binding, viral entry, and membrane fusion, whereas prM assists in folding, assembly, and function of the E protein [9, 11]. The ZIKV uses the envelope (E) glycoprotein for entry into specific cell types such as epidermal keratinocytes, fibroblasts, immature dendritic cells, and stem-cell-derived human neural progenitors [12]. Sequence comparisons of the E glycoprotein of ZIKV with the other members of the Flaviviridae family indicate an unusual degree of variability including glycosylation within the ZIKV strains [12]. These differences in glycosylation may determine a characteristic affinity for human target proteins.

Three major attachment factors for* Flavivirus* are heparin sulfates, dendritic cell-specific ICAM-3-grabbing nonintegrin 1 (DC-SIGN, CD209 antigen), and DC-SIGNR (CLEC4M), which interact with N-linked glycans of the viral E glycoprotein. DC-SIGN itself does not provide an essential internalization signal during DENV entry, suggesting that additional entry factors exist. It seems that ZIKV may also use GAGs (glycosaminoglycans) as attachment factors to enter the host cell [13].

Another molecule identified as an entry factor for DENV is AXL [14] which belongs to the TYRO3 AXL MER (TAM) family, a group of tyrosine kinase receptors involved in the clearance of apoptotic cells and regulation of innate immunity [15, 16]. However, for ZIKV, some studies confirm that AXL is not the key receptor for the viral infection of human neural progenitor cells [12, 17–20].

Receptor-mediated endocytosis is a prerequisite for fusion and uncoating of all known Flaviviridae family members. Clathrin-dependent uptake has been described as the major endocytosis mechanism, but alternative entry routes exist and may be used in a strain-specific manner [21].

After endocytic uptake and acidification of the endosomal lumen, the viral surface glycoproteins undergo a conformational change and induce fusion of the limiting endosomal membrane and the viral envelope. Disassembly of the viral capsid (“uncoating”) delivers the RNA genome to the cytoplasm, which completes the entry process [21].

After release of the genome into the cytoplasm, ZIKV replicates through a negative strand intermediate [9, 12]. For the nonstructural proteins synthesis and posttranslational modifications* Flavivirus* use the virus-induced membranous vesicles derived from the endoplasmic reticulum and Golgi complex by exploiting membrane trafficking [22].

Below we describe the current evidence of the “hijacking” of each of the nonstructural proteins with the host cell. Upon synthesis, the* Flavivirus* nonstructural proteins may play different functions within human host cells or may follow host exocytosis pathways and act outside the host cell. That is the case of the nonstructural protein NS1, a 46–55 KDa glycoprotein containing 2-3 glycosylation sites [23, 24]. After polyprotein processing, NS1 is translocated into the lumen of the ER and released from E protein by ER resident signal peptidase [23, 25]. The C-terminus is cleaved by an unidentified ER host protease [26] and glycosylated by the addition of high-mannose carbohydrates [27]. After a rapid dimerization, NS1 acquires a partially hydrophobic behaviour and can associate with cell membranes [11, 12, 28, 29]. NS1 protein can associate with the membrane through a glycosylphosphatidylinositol (GPI) anchor [30]. NS1 has also been described as being secreted to the extracellular environment [11, 28, 29]. The secreted form of NS1 traffics through the Golgi secretory pathway in mammalian cells, and the carbohydrate moieties are processed to more complex sugars that are then secreted as a soluble hexamer of ~300 kDa associated with lipids [11, 31–33].

NS2A is a multifunctional protein with roles in virion assembly [34, 35], RNA replication [36, 37], membrane permeation [38], and dissemination from infected mosquito midguts [8, 39, 40]. NS2A has also been shown to act as an interferon antagonist in different* Flavivirus* [8, 41, 42]. The NS2B protein interacts with NS3 to form a stable complex which functions as a serine protease [43] which has been shown to interfere with IFN-I induction [44]. ZIKV's NS3 protein contains a protease and a helicase domain that in several* Flavivirus* act independently of each other [45].

NS4B is an important IFN-I signaling antagonist during DENV2 infections by inhibiting the JAK/STAT pathway and antagonising STAT1 phosphorylation [46]. Unlike DENV, YFV NS4B blocks RIG-I through an interaction with STING. This highlights strain-specific variations used for IFN suppression between different* Flavivirus* [8].

NS5 offers some protection for the virus by producing capped viral RNA, enabling host RNA mimicry through its methyltransferase activity [47–50]. NS5 displays two enzymatic activities via the N-terminal methyltransferase domain and the C-terminal RNA dependent RNA polymerase (RdRp), which replicates viral RNA [51, 52].

Despite the description of receptors, entry factors or pathways for* Flavivirus* action, based on several experimental approaches, the specific cell surface receptor and endocytosis complexes, fusion mechanisms, and entry pathways for ZIKV, are not yet clear.

Although there is a similar genomic organization between ZIKV and DENV, nonstructural proteins exhibit low homology [53]. The recent publication of the spatial organization of the ZIKV proteins during the intracellular passage of the virus [54] and the high recombination frequencies seen in it suggest that ZIKV has potentially evolved faster and attained the ability to exploit multiple cell surface receptors and cellular factors to facilitate infection in a variety of cells types, differing from other* Flavivirus*. These evidences prompted an investigation of new ZIKV targets as a result of ZIKV specific PPIs established with the human host cell. Our approach was to use a computational-based analysis which is faster and more cost-effective than experimental methods and may be valuable for generating preliminary models.

This article presents information on potential Protein-Protein Interactions (PPIs) established between ZIKV structural and nonstructural proteins and human host proteins predicted by the OralInt algorithm [55]. The predicted PPIs are discussed considering the different mechanisms that have been proposed for* Flavivirus* and the intracellular localization of the viral proteins during the infection cycle.

## 2. Materials and Methods

This article aims at the clarification of the molecular entry and dissemination mechanisms of* Zika* virus by using a machine learning model for predicting the interactions established between the virus and the host proteins (PPI). These predictions are subsequently explored by a functional analysis based on the collection, organization, and interpretation of published information.

### 2.1. Human-*Zika* Virus Interactome Prediction

The prediction of the PPIs established between* Zika* virus and human proteins was performed using the OralInt tool developed by our group [55] which allows the prediction of interspecies PPIs. The input data were the human reviewed proteome (Proteome ID: UP000005640) (20199 proteins) and the ZIKV polyprotein sequence (Uniprot: Q32ZE1) using each of the processed proteins, both deposited in UniProt [65] as of January 2017. Of the 14 ZIKV proteins listed in UniProt, only the 10, which are currently considered functionally important, were used. Throughout this document, the proteins are identified by either their UniprotKB AC, gene, or protein name depending on the analysis performed.

The predicted interactions were stratified and analyzed according to the prediction score (0.9–1.0: very high confidence; 0.7–0.9: high confidence; 0.4–0.7: medium confidence; 0.1–0.4: low confidence). Interactions with scores lower than 0.1 were discarded. An Excel file with predicted PPIs used in this article is provided as supplementary material.

### 2.2. Visualization of PPI Network between Human and* Zika* Virus

A network of the predicted very high confidence PPIs (score ≥ 0.9) was generated using Cytoscape 3.5.0 [59]. To facilitate data interpretation, a network analysis was performed using the Network Analyzer Tool from Cytoscape, and visualization was obtained by mapping the node size onto degree (number of PPIs for each node) and the edge size onto score. An interactive network diagram created with the latest version of Cytoscape is included in the supplementary material.

### 2.3. Data of Protein Expression after* Zika* Virus Infection

To complement the functional analysis of the proteins involved in viral entry and virulence, data on the quantification of different proteins upon viral infection were used. As of March 2017, there were 2 studies with a large scale protein quantification in 2 different types of cell: (1) infected primary human fibroblasts [17] and (2) human cortical neural progenitors cells (hNPCs) [56]. In Hamel et al. 2015, the values represent fold inductions of mRNA copy numbers in infected cells relative to mock-infected cells and fold change values after 6 and 24 h are presented [17]. For hNPCs, fold change values were calculated from the log 2 values presented in the article using the inverse function *y* = 2^∧^*x* to ensure data standardization; this enables the comparison of the values obtained in the two studies. To facilitate interpretation, fold changes intervals were normalized by recalculating values between 0 and 1 as -(1/fold change). Protein quantification data used for discussion of results is available as an Excel file in the supplementary material.

### 2.4. Analysis of PPIs by Functional Role in* Zika* Virus Infection

For the study of the molecular mechanisms potentially involved in viral entry into the host, and subsequently intracellular affected mechanisms, an analysis of the predicted PPIs between the viral proteins and the host endocytosis receptors, immune response, and cytosolic host response proteins was performed. For this analysis, only the interactions with a score ≥ 0.2 were considered.

To verify which human proteins identified as having the potential to interact with the viral envelope and membrane proteins (score ≥ 0.2) have been described as membrane receptors involved in viral entry into the host cell, two approaches were followed:

(1) KEGG's [60, 61] mapping tool was used to identify proteins related to endocytosis.

(2) A review of the receptors involved in macropinocytosis of Flaviviridae [62] was used.

To evaluate how ZIKV modulates the host immune response, the PPIs established between the different ZIKV proteins and the host receptors and other proteins that have an effector or a signaling role in the immune response were analyzed. These data were integrated with data from expression fold change available for skin and hNPCs obtained as explained in Section 2.3 of the Material and Methods.

The initial sensing of infection is mediated by innate pattern recognition receptors (PRRs), which include Toll-like, RIG-I-like, NOD-like, and C-type lectin receptors. The intracellular signaling cascades triggered by these PRRs lead to transcriptional expression of inflammatory mediators that coordinate the elimination of pathogens and infected cells. To identify the molecular mechanisms used by ZIKV to bypass this defense system, PPIs predicted by OralInt between human and viral nonstructural proteins were considered.

VirHostNet 2.0 [63] complemented with the information present in ViralZone [64] were used to identify PRRs already described as being involved in ssRNA viruses recognition.

### 2.5. *Zika* and Dengue Virus Protein Homology Determination

The homology between the nonstructural ZIKV (strain Mr 766) and DENV (*Dengue* virus type 1 (strain Nauru/West Pac/1974) (DENV-1)) proteins was determined by using the Clustal Omega [66] algorithm provided as an Alignment Tool in UniProt [65].

## 3. Results and Discussion

### 3.1. Human-*Zika* Virus Predicted Interactome

Using OralInt [55], human-ZIKV interactome was determined and a summary of the results is presented in Table 1. The predicted PPIs are complemented with the annotation of the proteins which have been quantified in different human cells upon ZIKV infection. The quantification data pertain to transcriptomics data on the human proteins expressed by skin [17] and hNPCs cells [56] upon ZIKV infection. From a total of 1898 high to medium score (0.7–1) predicted PPIs, there are transcriptomics data on 726 of the human proteins involved. From these, the PPIs established between human and E and M ZIKV structural proteins are especially relevant for the identification of human target receptors.

Up to now, there is only one study that experimentally validates PPIs related to ZIKV infection [67]. OralInt predicts all of the 143 experimentally described interactions between NS2A and the human proteins and 33 of those are predicted with high or medium confidence.

Table 2 presents the number of human proteins interacting with a specific ZIKV protein, for which the PPIs have a score ≥0.4 and the annotation of the up- or downregulation of the human protein according to the values obtained in previous experiments reported in the literature [17, 56]. Since there has been interest in the identification and quantification of salivary biomarkers for this infection, proteins previously identified in saliva are also annotated [57, 58].

**Figure 1:**
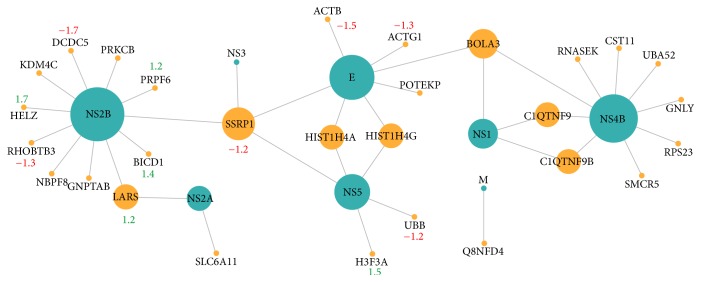
Network of OralInt predicted PPIs (score ≥ 0.9) established between ZIKV proteins (blue) and human proteins (orange). The size of the node denotes the degree (number of interactions established). Red denotes underexpressed and green overexpressed proteins. Expression data from Tang et al. 2016 [56]. Diagram generated with Cytoscape V3.5.0 [59].

The network of PPIs with a very high confidence score (≥0.9) is presented in Figure 1. No PPIs with the highest score were identified for the C and NS4A proteins. The viral proteins establishing the largest number of interactions are NS2B (protein which forms a complex with NS3 showing serine protease activity), NS4B, and E (the main protein binding to membrane receptors). Proteins with available quantification are also identified.

From the proteins interacting with the multifunctional ZIKV NS2B protein (Figure 1), the Rho-related BTB domain containing 3 (RHOBTB3) is of special note, since it is a Rab9-regulated ATPase required for vesicle transport and docking at the Golgi complex [68]. The prediction of this PPI with a high score is evidence that ZIKV interferes with vesicular organization and host docking mechanisms. Another protein establishing high score PPIs and involved in vesicular traffic is BICD1 (bicaudal D homolog 1 (Drosophila)) which regulates coat complex coatomer protein I- (COPI-) independent Golgi-endoplasmic reticulum transport by recruiting the dynein-dynactin motor complex [68]. Similarly, silencing and CRISPR/Cas9 knockout screens have previously identified another GTase Rab (RAB5C) and Rab-activating guanosine diphosphate/guanosine triphosphate exchange factors, GEFs (RABGEF), as vesicular transport factors contributing to* Flavivirus* effective invasion of the host cell [21].

### 3.2. New Endocytosis Pathway Targets Used by* Zika* Virus

Considering PPIs with a score ≥ 0.2 established between E and M ZIKV proteins and human membrane receptors, it is possible to identify potential entry mechanisms used by ZIKV.

Table 3 presents the receptors that ZIKV may use in both clathrin-dependent and independent pathways of endocytosis for infecting human cells. For each human to ZIKV E and M protein PPI, the respective score is presented. This information is completed with data from the quantification available in the literature [17, 56].

Regarding the use of receptors involved in clathrin-dependent endocytosis by ZIKV, the greater scores are for PPIs established with the GRPCR and RTK type receptors. The interaction of ZIKV E protein with the beta-2 adrenergic receptor (ADRB2) of the GRPCR family, which mediates the catecholamine-induced activation of adenylate cyclase through the action of G proteins, is predicted with a score of 0.4. The PPI established between the ZIKV M protein and the ADRB3, a beta-3 adrenergic receptor, also has a 0.4 score. The RTK receptor type, namely, hepatocyte growth factor receptor (MET), which during embryonic development has a role in gastrulation, development, and migration of muscles and neuronal precursors, angiogenesis, and kidney formation, establishes with ZIKV E protein a PPI with 0.4 score.

Belonging to the GRPCR type receptors, the CCR5 (chemokine C-C motif receptor 5) and CXCR4 (chemokine C-X-C motif receptor 4) both establish interactions with ZIKV E protein having a score of 0.2.

From the RTK type receptors, ERBB4, a tyrosine-protein kinase that plays an essential role as cell surface receptor for neuregulins, together with the EGF family members, regulates development including the central nervous system. Therefore, they are worthy of special note due to their potential impact in central nervous system development.

Table 3 identifies 10 receptors for clathrin-independent endocytosis. AXL receptor which belongs to the TYRO3 AXL MER (TAM) family, a group of tyrosine kinase receptors involved in the clearance of apoptotic cells and regulation of innate immunity [15, 16], is the best described target of* Flavivirus*. It has been shown that AXL is also the primary ZIKV entry cofactor on human umbilical vein endothelial cells (HUVECs) and that ZIKV uses AXL with much greater efficiency than DENV or WNV, by binding the AXL ligand GAS6 which recognizes phosphatidylserine (PS) exposed at the surface of the viral envelope and bridges the viral particle binding to the AXL receptor. This mechanism of viral entry, based on PS exposure, has been termed viral apoptotic mimicry [62]. From OralInt's results we can conclude that E ZIKV protein establishes a PPI with AXL with a 0.2 score, having the potential of interacting also with GAS6 with a score of 0.1. With M ZIKV protein, GAS6 establishes a PPI with a score of 0.2.

Once AXL is activated, it mediates signaling through its tyrosine kinase domain to dampen type I interferon (IFN1) signaling and facilitate infection [14, 69, 70]. Since AXL is expressed on primary human placental cells, endothelial cells, fibroblast cells, amniotic epithelial cells, trophoblast progenitors, and macrophages (Hofbauer cells) the maternal-fetal transmission of ZIKV is facilitated [12, 71–73]. AXL was recently shown to support ZIKV infection of human foreskin fibroblasts [17] and its expression was noted in the brain and neural progenitor cells [74–76].

According to our PPI scores, the TIM type receptor was the highest for the ZIKV clathrin-independent endocytosis mechanisms. Within that group of receptors, the hepatitis A virus cellular receptor 2 (HAVCR2) establishes a PPI with a 0.4 score with ZIKV protein E. However, other receptors also seem to interact with ZIKV proteins, namely, the caveolin-3 (CAV3) which establishes a PPI with M protein having a score of 0.6. Caveolins like CAV2 and CAV3 act as scaffolding proteins within caveolar membranes that interact directly with G-protein alpha subunits and can functionally regulate their activity. Internalization via caveolae is not a constitutive process but only occurs upon cell stimulation. It has been described that caveosomes participate in the transport of the simian virus 40 and other pathogens from the cell surface to the endoplasmic reticulum [77]. Caveolin-2 is most prominently expressed in fibrous and adipose tissue and caveolin-3 is restricted to striated and smooth muscle. We hypothesize that this may be another pathway through which maternal-fetal transmission occurs.

The M protein of ZIKV also establishes a PPI with CD81 with a 0.4 score. Both E and M proteins establish PPIs with claudin-1 (CLDN1) with a score of 0.3 and it has been shown that the expression of this protein had a 2.5-fold increase upon ZIKV infection. CLDN1 plays a major role in tight junction-specific obliteration of the intercellular space through calcium-independent cell-adhesion activity that regulates the permeability of epithelia. Claudin-1 and CD81 have also been related to the HCV entry into host cell [60, 61].

It is known that membrane proteins when interacting with other proteins (cognate ligands) are subject to conformational changes. We think that the same happens when the E ZIKV structural protein binds to the host membrane receptors and causes the exposure of M protein interaction domains. It has been described that* Flavivirus* structural proteins assume many asymmetric states [78] and are in continuous dynamic motion [79], which likely exposes patches of the virion membrane [80]. Both facts would explain why ZIKV M protein might interact with the host cell receptors showing a high score, as what happens in the PPIs established with CAV3 and CD81.

Additionally, Table 3 shows the scores of PPIs established between ZIKV and C-type lectins dendritic cell-specific intercellular adhesion molecule-3-grabbing nonintegrin (DC-SIGN), a pathogen-recognition receptor expressed on the surface of immature dendritic cells involved in initiation of primary immune response that mediates the endocytosis of pathogens [17], and DC-SIGN-related protein (L-SIGN). Both E and M ZIKV proteins establish PPIs with a score 0.2 with DC-SIGN and with the C-type lectin domain family 4 member M (CLEC4M), a L-SIGN type receptor for mannose-like carbohydrates [81].

### 3.3. New Immune Targets Used by* Zika* Virus

ZIKV modulation of the immune response mechanisms may be seen as an action controlled by the viral E and M structural proteins which bind and activate human receptors or interact with other membrane proteins or even bind extracellular proteins impairing their action. Once the nonstructural proteins are produced they may exert their function by binding and/or modifying the host proteins available. Protein synthesis of ZIKV proteins follows the endomembrane trafficking system in a similar fashion as to what happens with the host proteins up to the Golgi complex where glycosylation ends. This whole pathway has been demonstrated for NS1 and this nonstructural protein may also be secreted just as the host proteins and exert its actions in extracellular compartments.

In Table 4, PPIs established between different ZIKV proteins and host receptors and other proteins with an effector or a signaling role in the immune response are presented. Additionally, information relative to the fold change is annotated for skin and hNPCs when available [17, 56].

IFN and TLRs receptors are important to convey signals to the cell and initiate antiviral defense mechanisms. Whether ZIKV is able to bind IFN receptors is still not clear. OralInt predicts PPIs between ZIKV E protein and IFNAR1/IFNAR2 receptor subunits, with scores 0.3 and 0.2, respectively (Table 4). After type I IFN binding to IFN receptor, the signal pathway leads to the induction of an antiviral state [13, 82].

The PPI scores with ZIKV E protein and TLR2 or TLR4 are 0.3. ZIKV E and M proteins can both interact with TLR6 (0.2 score).

It has been demonstrated that nonstructural proteins of* Flavivirus* may interact with TLR receptors. In the case of DENV, it was shown that NS1 (probably the soluble hexamer) binds TLR4 on the surface of CD14+ monocytes and induces cellular activation, cytokine production, and vascular permeability, a similar response triggered by the bacterial LPS [11]. The results presented in Table 4 show that all ZIKV proteins, except NS3, establish PPIs with TLRs with scores ranging from 0.2 to 0.5.

Complement proteins are an important part of the innate immune response and as signaling molecules for different types of immune cells.

DENV NS1 can attenuate activation of the classical, lectin, and alternative pathways by interacting with complement proteins and their regulators [83].

C8B, a constituent of the membrane attack complex (MAC) that plays a key role in the innate and adaptive immune response by forming pores in the plasma membrane of target cells, is the protein that establishes PPIs with the highest scores with ZIKV proteins. M ZIKV protein interacts with C6 and C8B with scores of 0.7 and 0.6, respectively.

Cytokines and chemokines may interact with structural and nonstructural ZIKV proteins (Table 4) with scores of 0.2–0.7 for both.

### 3.4. New Immune Modulation Pathway Targets Used by* Zika*

The coevolution between* Flavivirus* and their hosts has taken place over a long period. Host cells have developed multiple branches of innate immune system to keep the virus invasion and replication under control [84]. Conversely the viruses have developed different mechanisms to evade the induction of an antiviral state by the host cell and in some cases the prevention of the triggering of the apoptotic state of the host cell. A synergistic effect of nonstructural proteins to restrict cellular antiviral responses at multiple levels has been demonstrated [13].

**Figure 2:**
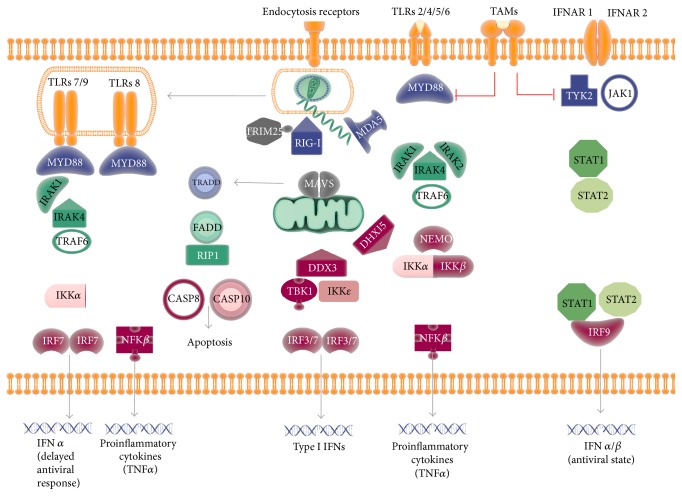
Diagram representing membrane and cytosolic targets of ZIKV used for host cell entry and immune response modulation. Information was obtained from OralInt predicted PPIs and the literature [60–64].

Table 5 presents the predicted PPIs established between the nonstructural proteins of ZIKV and proteins of the host pathways leading to an antiviral state (IFNs) and proinflammatory cytokine (TNF*α*) synthesis which are depicted in Figure 2. The integration of fold change data in skin and hNPCs in Figure 2 allows the evaluation of the pathways which may occur in these two cell types during ZIKV infection.

Several PRRs have been demonstrated as being activated for different* Flavivirus*. These include Toll-like receptors (TLR) mediated responses, specific nucleic acid receptor activation such as RIG-I and PKR, and the mitochondrial antiviral immunity (MAV) and IFN receptors dependent pathways. Figure 2 also integrates the signaling pathways dependent on endocytosis receptors which were previously discussed (Table 3).

The detection of cytoplasmic viral RNA [85] is accomplished by RLRs as DDX58 (the retinoic acid inducible gene-1) (RIG-I), a RNA helicase that recognizes viral RNA present within the cytoplasm and melanoma differentiation-associated protein 5 (MDA5) [86].

RIG-I recognizes short RNA ligands with 5′-triphosphate caps. MDA5 recognizes long kilobase-scale genomic RNA and replication intermediates. Ligand binding induces conformational changes and oligomerization of RLRs that activate the signaling partner MAVS on the mitochondrial and peroxisomal membranes. This signaling process is under tight regulation, dependent on posttranslational modifications of RIG-I and MDA5. Both contain a helicase domain and a C-terminal domain, which are involved in the binding of viral RNA. This then signals through IRF3/7 activating the transcription of IFNs [87].

The RIG-I molecule is upregulated with a fold change of 4.9 after 24 hours of infection of fibroblasts and of the TRIM25 an E3 ubiquitin ligase, which further activates RIG-I (Table 5). TRIM25 functions as an E3 ligase, which adds poly-ubiquitin chains to the amino-terminal of RIG-I [87]. This is thought to facilitate the interaction of RIG-I with MAVS, thus modulating downstream signaling of the IFN-I response.

RIG-I has been demonstrated as modulating DENV antiviral response [88]. Through the direct interaction and modulation of I*κ*B kinase *ε*, an important kinase involved in IFN-I induction, DENV NS2B/NS3 disrupts RIG-I, the signaling pathway.

ZIKV nonstructural proteins establish PPIs with scores of 0.2 with RIG-I, whereas PPIs established with TRIM25 and MDA5 have scores between 0.3 and 0.6. The RNA sensing mechanism MDA5, also known as interferon induced with helicase C domain 1 [88] upon ZIKV infection of fibroblasts, has a similar variation to RIG-I, decreasing initially and being upregulated with a fold change of 7.3 after 24 hours (Table 5). This evidence points to a delayed cell response, which seems to be dependent on the presence of several ssRNA molecules which only happens after the virus initiated replication within the host cell.

Recently, it has been shown that when ZIKV infects the primary human placental macrophages and placental cytotrophoblasts, it induces the production of IFN-*α*, proinflammatory cytokines, and antiviral genes such as RIG-I and MDA5 [89]. Also during infection, ZIKV stimulates cell death and induces type I interferon (IFN) response and proinflammatory cytokines that disrupt the placental barrier leading to neurological disorders such as microcephaly [74].

The IFNs bind to a heterodimeric transmembrane receptor which results in the recruitment and activation of tyrosine kinases, JAK1 and TYK2, through auto- and transphosphorylation. This process drives the recruitment and subsequent phosphorylation of the cytoplasmic transcription factors, STAT1 and STAT2, which translocate to the nucleus and associate with IRF9 to activate IFN genes.

NS5 has been described as a potent* Flavivirus* IFN-I antagonist [90] by STAT1/2 activation or translocation. DENV NS5 binds and degrades STAT2 by targeting it for ubiquitin-mediated proteasomal degradation [91].

NS2B and NS4B from ZIKV establish PPIs with a 0.4 score with JAK1 and a score of 0.3 with TYK2. Both tyrosine kinases, JAK1 and TYK2, are upregulated in hNPCs under ZIKV infection (Table 5). All the nonstructural ZIKV proteins establish PPIs with STAT1 (scores 0.5–0.8). Similarly, experimental studies have confirmed that ZIKV NS5 is required for the proteasomal degradation of the STAT2 in humans [92, 93]. However, OralInt's score for STAT2 PPIs established with viral proteins is only 0.3. We propose that STAT1 may be a potential ZIKV target.

During* Flavivirus* infections TLR7, TLR8, TLR9, and the dimerization complex TLR7 with TLR9, which identify RNA, are important factors for virus detection. All TLRs mentioned signal through an intermediate protein, MYD88, which eventually leads to activation of the nuclear factor kappa-B (NFKB), a pleiotropic transcription factor present in almost all cell types. One of the cytokines which results from the activation of this pathway, TNF-alpha, is downregulated (Table 5) which indicates that, despite being noticed by the cell, the virus somehow inhibits, at least temporarily, a systemic inflammatory response by avoiding the release of proinflammatory cytokines.

NS1 and NS2A ZIKV proteins can interact with MYD88 (0.4 score). NS4B establishes a PPI with a score of 0.3 with MYD88. Both NS2B and NS5 interact with MYD88 with a score of 0.4. Curiously, NS2B, the serine protease of ZIKV, can interact with NFKB with a score of 0.4 and may result in the degradation of the transcription factor by the activity of the viral protease.

It was recently demonstrated that IFN*β* restricts replication of ZIKV and promotes autophagic degradation of NS2B/NS3 complex, which explains the host innate immune protective defense against ZIKV. As the ubiquitination of NS2B/NS3 is enhanced by IFN*β* treatment and STAT1 is required for the degradation of NS2/NS3, the potential IFN-inducible E3 ligases might be involved in this process. Many E3 ligases such as tripartite motif (TRIM) proteins family members, including TRIM25, can be upregulated by IFN through STAT1 [94].

The PPIs predicted by OralInt show high scores for interaction of TRIM25 (NS2B—0.4). The PPI score between IFN*β* and NS2B is 0.2 and with NS3 is 0.3.

The fact that there is a low homology between the nonstructural DENV and ZIKV proteins, as determined by the UniProt Alignment Tool [65], especially for NS1 (54%), NS2A (24%), NS2B (37%), NS3 (65%), and NS5 (65%), further supports the search for different targets and the establishment of different PPIs than those described for DENV.

Although ZIKV uses many of the main pathways exploited by other* Flavivirus* to infect human cells, which is represented in the PPIs predicted by OralInt, new ZIKV targets are possible within the same general pathways based on higher score of the PPIs obtained.

## 4. Conclusion

**Figure 3:**
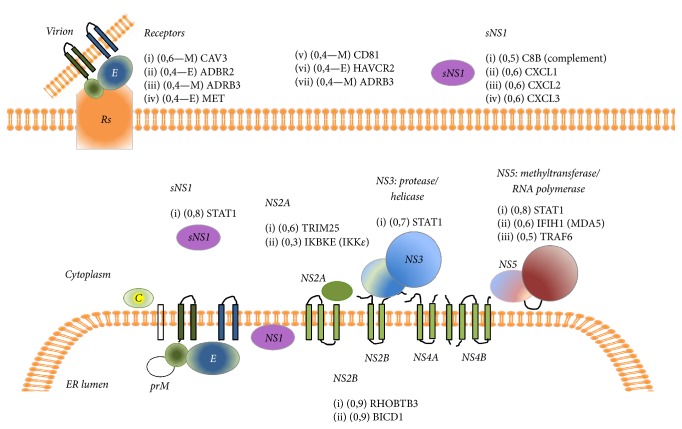
Summary diagram of the proposed ZIKV targets considering the intra- and extracellular localization of the viral proteins during the infection cycle. Scores of the OralInt predicted PPIs are presented next to each human protein target and refer to interaction with the specific ZIKV protein. The proposed membrane orientation of the ZIKV proteins was modified from Figure 25.4 in Shi and Gao (2017) [54].

The analysis of the ZIKV-human interactome reveals that this virus shares some of the targets and strategies with other* Flavivirus* to infect human host cells. However, we found new interactions that support the existence of different human protein targets which may be used specifically by ZIKV to invade and disrupt the host cell homeostasis (Figure 3). Despite having a similar genome organization as other* Flavivirus,* the low homology between ZIKV and DENV nonstructural proteins justifies the analysis and in silico search for new targets and we believe that these are worthy of further attention. The computational approach for the discovery of new targets and mechanisms of ZIKV-human infection is an expedite and efficient way of making new proposals which should be experimentally confirmed by quantitative proteomics analysis enabling the development of innovative preventive (vaccines) or therapeutic approaches.

## Figures and Tables

**Table 1:** Number of interactions between human and ZIKV proteins predicted by OralInt according the score (0.9–1: very high confidence; 0.7–0.9: high confidence; 0.7–0.4: medium confidence). Quantification refers to the number of human proteins which have been identified as being expressed upon ZIKV infection of fibroblast (skin) and human cortical neuronal progenitor cells (hNPCs).

*Zika* protein	OralInt score 0.9–1		OralInt score 0.7–0.9		OralInt score 0.4–0.7	
	PPI	Quantified hNPCs^*∗*^	PPI	Quantified hNPCs^*∗*^ + Skin^*∗∗*^	PPI	Quantified hNPCs^*∗*^ + Skin^*∗∗*^
Capsid (C)	-	-	168	64	3236	1055 + 14 (4)
Envelope (E)	7	3	124	42 + 1	2757	838 + 11 (3)
Membrane (M)	1	-	67	15	1314	369 + 3
NS1	3	-	119	39 + 1	2612	824 + 10 (3)
NS2A	2	1	264	81 + 1	4478	1486 + 17 (6)
NS2B	11	7	439	148 + 2	4649	1604 + 18 (4)
NS3	1	1	33	12 + 1	979	304 + 3 (1)
NS4A	-	-	124	41 + 1	2618	840 + 12 (3)
NS4B	9	-	292	83 + 1	3858	1189 + 12 (4)
NS5	5	2	229	78 + 1	3970	1330 + 17 (5)

*Total*	*39*	*14*	*1859*	*603 + 9*	*30471*	*9869 + 103*

^**∗**^[56]; ^**∗∗**^[17].

**Table 2:** Number of human proteins establishing PPIs exclusively with each ZIKV protein; human proteins expression regulation upon ZIKV infection (when available) and respective presence in saliva. Only PPIs with scores ≥ 0.4 are shown.

*Zika* protein	Total PPIs	Human proteins with expression data^*∗*^		Proteins present in saliva^*∗∗*^
		Upregulated	Downregulated	
E	105	16	21	8
M	62	8	9	3
NS1	131	16	14	4
NS2A	638	85	94	22
NS2B	1066	214	175	45
NS3	1	-	1	-
NS4A	46	5	7	3
NS4B	300	37	43	8
NS5	557	90	106	22

^**∗**^Quantification in human cortical neural progenitor cells (hNPCs) [56] and skin (fibroblasts) [17]. ^**∗∗**^Presence in saliva from OralCard [57, 58].

**Table 3:** Membrane receptors as potential targets for ZIKV entry into host cells. Only proteins establishing PPIs with scores ≥ 0.2 are presented (OralInt prediction).

Endocytosis receptors	Type of receptor	Gene name	UniprotKB AC	PPI score		Fold change hNPCs^*∗*^
				E	M	
Clathrin-dependent endocytosis	GRPCR	ADRB2	P07550	0.4		3.6
		ADRB3	P13945	0.3	0.4	
		ADRB1	P08588	0.2	0.2	
		CCR5	P51681	0.2		
		CXCR4	P61073	0.2		
	LDLR	LDLR	P01130	0.2		1.2
	RTK	EGFR	P00533	0.3		
		ERBB4	Q15303	0.2		
		FGFR2	P21802	0.2		1.7
		FGFR3	P22607		0.2	−1.5
		FGFR4	P22455	0.2		
		MET	P08581	0.4		
		PDGFRA	P16234	0.3		
	TFR	TFRC	P02786		0.2	
	TGFBR	TGFBR2	P37173	0.3	0.3	
		TGFBR3	Q03167	0.3		
		TGFBR1	P36897	0.2		

Clathrin-independent endocytosis	Caveolin	CAV2	P51636	0.2	0.2	
		CAV3	P56539	0.2	0.6	
	Others	CD81	P60033	0.3	0.4	−1.2
		CLDN1	O95832	0.3	0.3	2.5
		IL2RG	P31785	0.3		
		OCLN	Q16625			−1.3
	TIM	HAVCR2	Q8TDQ0	0.4	0.2	
	TAM	AXL	P30530	0.2		1.3
	DC-SIGN	CD209	Q9NNX6	0.2	0.2	
	L-SIGN	CLEC4M	Q9H2X3	0.2	0.2	

^*∗*^Human cortical neural progenitor cells (hNPCs) [56].

**Table 4:** PPIs established between the different ZIKV proteins and cell receptors and other proteins with an effector or signalling role in the immune response. Only proteins establishing PPIs with scores ≥ 0.2 are presented (OralInt prediction).

Pathway	Gene Name	UniprotKB AC	OralInt score									Fold change hNPCs^*∗*^	Fold change skin 6 h^*∗∗*^	Fold change skin 24 h^*∗∗*^
			E	M	NS1	NS2A	NS2B	NS3	NS4A	NS4B	NS5			
IFN receptor	IFNAR1	P17181	0.3		0.2	0.2	0.3		0.2		0.3	−1.3	−1.4	−1.3
	IFNAR2	P48551	0.2				0.2				0.2			

Toll-like receptor	TLR2	O60603	0.3		0.3	0.3	0.2		0.2	0.4	0.3			
	TLR4	O00206	0.3		0.2	0.4	0.3		0.2	0.3	0.2			
	TLR5	O60602												
	TLR6	Q9Y2C9	0.2	0.2		0.2	0.5		0.4	0.3	0.2			

Complement	C2	P06681	0.2	0.3			0.3	0.3	0.2					
	C3	P01024				0.3			0.5	0.5	0.4			
	C4A	P0C0L4	0.2	0.2			0.2	0.2	0.3	0.3	0.4			
	C4B	P0C0L5	0.2	0.2			0.2	0.2		0.3	0.3			
	C5	P01031	0.2	0.3	0.3	0.3	0.3	0.4	0.2	0.2				
	C6	P13671	0.3	0.7	0.2	0.5	0.5	0.4		0.2	0.2			
	C7	P10643	0.3	0.2		0.2	0.2			0.2	0.2			
	C8A	P07357		0.3		0.4	0.3	0.3	0.4	0.3	0.3			
	C8B	P07358	0.5	0.6	0.5	0.4	0.5	0.6	0.4	0.5	0.6			
	C8G	P07360	0.3	0.4	0.2	0.3	0.5	0.3	0.3	0.5	0.3			
	C9	P02748		0.2		0.2			0.3	0.0				

Cytokines	IL1B	P01584	0.3		0.3	0.4	0.2	0.2	0.2	0.3	0.4		−3.2	−5.3
	IL6	P05231	0.4	0.3	0.4	0.2		0.3	0.2	0.4	0.4		1.1	8.2
	IL17	Q16552	0.4	0.6	0.4	0.2	0.5	0.3	0.4	0.6	0.4			
	IL17F	Q96PD4		0.2			0.2		0.2					
	IL23A	Q9NPF7		0.2			0.4		0.2					
	IL31	Q6EBC2	0.4	0.4	0.3	0.2	0.3	0.3	0.2	0.4	0.4			
	CD40L	P29965	0.2			0.3		0.2	0.2	0.2	0.2			
	IFNG	P01579	0.2				0.4		0.5		0.2			
	TNFA	P01375				0.2							−1.7	−2.1
	IL22	Q9GZX6								0.2				
	IL21	Q9HBE4		0.3			0.3		0.3	0.3				
	IL33	O95760			0.2						0.2			
	IL4	P05112	0.3	0.5	0.4	0.3	0.3	0.2	0.4	0.7	0.2			
	IL10	P22301	0.2	0.2	0.2	0.2	0.3	0.2	0.2	0.3	0.2			
	IL25	Q9H293	0.3		0.3	0.2	0.2		0.2	0.3	0.3			

Chemokines	CCL2 (MCP-1)	P13500												
	CCL3 (MIP-1a)	P10147	0.2	0.2	0.2	0.2	0.4	0.2		0.2	0.2		−1.7	−1.4
	CCL4 (MIP-1*β*)	P13236	0.4	0.2	0.3	0.3	0.5	0.4	0.4	0.4	0.5			
	CXCL1 (Gro-a)	P09341	0.6	0.5	0.6	0.6	0.7	0.4	0.5	0.7	0.4			
	CXCL2 (Gro-*β*)	P19875	0.6	0.5	0.6	0.6	0.7	0.4	0.5	0.7	0.4			
	CXCL3	P19876	0.6	0.5	0.6	0.6	0.7	0.4	0.5	0.7	0.4			
	CXCL8 (IL-8)	P10145	0.2	0.5	0.3	0.3	0.5		0.5	0.5	0.2		−2.8	3.7

^*∗*^Human cortical neural progenitor cells (hNPCs) [56]. ^*∗∗*^Skin (fibroblasts) [17].

**Table 5:** PPIs established between the nonstructural proteins of ZIKV and proteins of the host pathways leading to an antiviral state. Only proteins establishing PPIs with scores ≥ 0.2 are presented (OralInt prediction).

Pathway	Gene name	UniprotKB AC	PPI score with NS1	PPI score with NS2A	PPI score with NS2B	PPI score with NS3	PPI score with NS4A	PPI score with NS4B	PPI score with NS5	Fold change hNPCs	Fold change skin 6 h	Fold change skin 24 h
IFN receptor	IRF9	Q00978		0.2					0.2			
	JAK1	P23458	0.2	0.3	0.4		0.3	0.4	0.3	1.3		
	TYK2	P29597	0.3	0.3	0.3		0.2	0.3	0.2	1.5		

Toll-like receptor	TNF	P01375		0.2							−1.7	−2.1
	IRAK1	P51617		0.2	0.2		0.2	0.2	0.2	−1.3	1.4	1.6
	IRAK2	O43187	0.3	0.3	0.3			0.3	0.2			
	IRAK4	Q9NWZ3		0.2	0.2				0.2			
	IKBKG (NEMO)	Q9Y6K9	0.3	0.2	0.2		0.2	0.3	0.2			
	MYD88	Q99836	0.4	0.4	0.2		0.2	0.3	0.2	−1.8	−1.0	1.8
	NFKB	P19838	0.2	0.3	0.4	0.2	0.2	0.2	0.4	1.3	1.2	1.6
	TLR7	Q9NYK1									−1.7	−1.4
	TLR8	Q9NR97									−1.7	−1.4
	TLR9	Q9NR96									−1.0	−2.3
	TBK1	Q9UHD2		0.2	0.2					1.6	−1.2	1.1

RIG-like receptor	DDX3X (DDX3)	O00571			0.2						1.3	−1.1
	IKBKE (IKK*ε*)	Q14164		0.3	0.4		0.2	0.2				
	TRIM25	Q14258	0.4	0.6	0.4		0.3	0.4	0.4		1.4	1.8
	STAT1	P42224	0.8	0.5	0.7	0.7	0.6	0.6	0.8		1.1	1.8
	STAT2	P52630	0.3	0.3			0.2	0.3	0.2			
	DDX58 (RIG-I)	O95786	0.2	0.2	0.2					2.0	−3.2	4.9

PKR-RNA and stress sensors (apoptosis modulation)	CASP8	Q14790									2.0	1.3
	CASP10	Q92851							0.2		3.3	3.3
	FADD	Q13158						0.2	0.1	1.5	−1.3	−1.1
	RIPK1 (RIP1)	Q13546	0.2	0.2	0.5		0.3	0.3	0.2		1.6	1.4
	TRADD	Q15628									1.5	1.3

Mitochondria antiviral immunity	IFNA1 (IFN*α*)	P01562					0.2	0.2			2.3	3.4
	IFNB1 (IFN*β*)	P01574	0.3		0.2	0.3	0.2	0.3	0.4		−1.7	3.7

Toll/mitochondria antiviral immunity	TRAF6	Q9Y4K3	0.4	0.5	0.2	0.2	0.2	0.4	0.5	1.3	1.3	−1.1
	IFIH1 (MDA5)	Q9BYX4	0.4	0.5	0.4	0.4	0.4	0.3	0.6		−1.4	7.3

RIG/mitochondria antiviral immunity	IRF3	Q14653		0.3				0.2	0.2		−1.1	−1.1
	MAVS	Q7Z434								−1.3	−1.0	−1.5

Toll/RIG/mitochondria antiviral immunity	CHUK (IKK*α*)	O15111	0.3	0.3	0.4	0.2	0.4	0.2	0.3	1.3	1.0	1.2
	IKBKB (IKK*β*)	O14920	0.2	0.4	0.6		0.4	0.2	0.2	−1.9	1.7	1.1
	IRF7	Q92985	0.2		0.2		0.2	0.2	0.3	1.6	1.3	3.2

^*∗*^Human cortical neural progenitor cells (hNPCs) [56]. ^*∗∗*^Skin (fibroblasts) [17].

## Supplementary Materials

Supplementary Materials Excel file with Protein-Protein Interactions (PPIs) between human and ZIKV, predicted in this work and respective scores. Excel file with the protein quantification data, obtained from the literature, used in the discussion of the results. Interactive network diagram created with Cytoscape (.CYS) freeware software downloadable at cytoscape.org [http://cytoscape.org].

## References

[1] Cao-Lormeau V.-M., Blake A., Mons S. (2016). Guillain-barré syndrome outbreak associated with zika virus infection in french polynesia: a case-control study. *The Lancet*.

[2] De Oliveira C. S., Da Costa Vasconcelos P. F. (2016). Microcephaly and Zika virus. *Jornal de Pediatria*.

[3] Fauci A. S., Morens D. M. (2016). Zika virus in the americas—yet another arbovirus threat. *The New England Journal of Medicine*.

[4] Gould E. A., Solomon T. (2008). Pathogenic flaviviruses. *The Lancet*.

[5] Mackenzie J. S., Gubler D. J., Petersen L. R. (2004). Emerging flaviviruses: the spread and resurgence of Japanese encephalitis, West Nile and dengue viruses. *Nature Medicine*.

[6] Oehler E., Watrin L., Larre P. (2014). Zika virus infection complicated by Guillain-Barré syndrome – case report, French Polynesia, December 2013. *Eurosurveillance*.

[7] Ventura C. V., Maia M., Bravo-Filho V., Góis A. L., Belfort R. (2016). Zika virus in Brazil and macular atrophy in a child with microcephaly. *The Lancet*.

[8] Cumberworth S. L., Clark J. J., Kohl A., Donald C. L. (2017). Inhibition of type I interferon induction and signalling by mosquito-borne flaviviruses. *Cellular Microbiology*.

[9] Lindenbach B. D., Rice C. M. (2003). Molecular biology of flaviviruses. *Advances in Virus Research*.

[10] Mukhopadhyay S., Kuhn R. J., Rossmann M. G. (2005). A structural perspective of the Flavivirus life cycle. *Nature Reviews Microbiology*.

[11] Conde J. N., Silva E. M., Barbosa A. S., Mohana-Borges R. (2017). The complement system in flavivirus infections. *Frontiers in Microbiology*.

[12] Routhu N. K., Byrareddy S. N. (2017). Host-Virus Interaction of ZIKA Virus in Modulating Disease Pathogenesis. *Journal of Neuroimmune Pharmacology*.

[13] Wu Y., Liu Q., Zhou J. (2017). Zika virus evades interferon-mediated antiviral response through the co-operation of multiple nonstructural proteins in vitro. *Cell Discovery*.

[14] Meertens L., Carnec X., Lecoin M. P. (2012). The TIM and TAM families of phosphatidylserine receptors mediate dengue virus entry. *Cell Host & Microbe*.

[15] Lemke G., Rothlin C. V. (2008). Immunobiology of the TAM receptors. *Nature Reviews Immunology*.

[16] Rothlin C. V., Ghosh S., Zuniga E. I., Oldstone M. B. A., Lemke G. (2007). TAM Receptors Are Pleiotropic Inhibitors of the Innate Immune Response. *Cell*.

[17] Hamel R., Dejarnac O., Wichit S. (2015). Biology of Zika virus infection in human skin cells. *Journal of Virology*.

[18] Garcez P. P., Loiola E. C., Da Costa R. M. (2016). Zika virus impairs growth in human neurospheres and brain organoids. *Science*.

[19] Shao Q., Herrlinger S., Yang S.-L. (2016). Zika virus infection disrupts neurovascular development and results in postnatal microcephaly with brain damage. *Development*.

[20] Wells M. F., Salick M. R., Wiskow O. (2016). Genetic Ablation of AXL Does Not Protect Human Neural Progenitor Cells and Cerebral Organoids from Zika Virus Infection. *Cell Stem Cell*.

[21] Gerold G., Bruening J., Weigel B., Pietschmann T. (2017). Protein interactions during the Flavivirus and hepacivirus life cycle. *Molecular & Cellular Proteomics*.

[22] Miller S., Krijnse-Locker J. (2008). Modification of intracellular membrane structures for virus replication. *Nature Reviews Microbiology*.

[23] Muller D. A., Young P. R. (2013). The flavivirus NS1 protein: molecular and structural biology, immunology, role in pathogenesis and application as a diagnostic biomarker. *Antiviral Research*.

[24] Edeling M. A., Diamond M. S., Fremont D. H. (2014). Structural basis of flavivirus NS1 assembly and antibody recognition. *Proceedings of the National Acadamy of Sciences of the United States of America*.

[25] Zhang F., Hammack C., Ogden S. C. (2016). Molecular signatures associated with ZIKV exposure in human cortical neural progenitors. *Nucleic Acids Research*.

[26] Falgout B., Markoff L. (1995). Evidence that flavivirus NS1-NS2A cleavage is mediated by a membrane- bound host protease in the endoplasmic reticulum. *Journal of Virology*.

[27] Pryor M. J., Wright P. J. (1994). Glycosylation mutants of dengue virus NS1 protein. *Journal of General Virology*.

[28] Winkler G., Maxwell S. E., Ruemmler C., Stollar V. (1989). Newly synthesized dengue-2 virus nonstructural protein NS1 is a soluble protein but becomes partially hydrophobic and membrane-associated after dimerization. *Virology*.

[29] Schlesinger J. J., Brandriss M. W., Putnak J. R., Walsh E. E. (1990). Cell surface expression of yellow fever virus non-structural glycoprotein NS1: Consequences of interaction with antibody. *Journal of General Virology*.

[30] Jacobs M. G., Robinson P. J., Bletchly C., Mackenzie J. M., Young P. R. (2000). Dengue virus nonstructural protein 1 is expressed in a glycosyl-phosphatidylinositol-linked form that is capable of signal transduction. *The FASEB Journal*.

[31] Crooks A. J., Lee J. M., Dowsett A. B., Stephenson J. R. (1990). Purification and analysis of infectious virions and native non-structural antigens from cells infected with tick-borne encephalitis virus. *Journal of Chromatography A*.

[32] Flamand M., Megret F., Mathieu M., Lepault J., Rey F. A., Deubel V. (1999). Dengue virus type 1 nonstructural glycoprotein NS1 is secreted from mammalian cells as a soluble hexamer in a glycosylation-dependent fashion. *Journal of Virology*.

[33] Gutsche I., Coulibaly F., Voss J. E. (2011). Secreted dengue virus nonstructural protein NS1 is an atypical barrel-shaped high-density lipoprotein. *Proceedings of the National Acadamy of Sciences of the United States of America*.

[34] Kümmerer B. M., Rice C. M. (2002). Mutations in the yellow fever virus nonstructural protein NS2A selectively block production of infectious particles. *Journal of Virology*.

[35] Leung J. Y., Pijlman G. P., Kondratieva N., Hyde J., Mackenzie J. M., Khromykh A. A. (2008). Role of nonstructural protein NS2A in flavivirus assembly. *Journal of Virology*.

[36] MacKenzie J. M., Khromykh A. A., Jones M. K., Westaway E. G. (1998). Subcellular localization and some biochemical properties of the flavivirus Kunjin nonstructural proteins NS2A and NS4A. *Virology*.

[37] Rossi S. L., Fayzulin R., Dewsbury N., Bourne N., Mason P. W. (2007). Mutations in West Nile virus nonstructural proteins that facilitate replicon persistence in vitro attenuate virus replication in vitro and in vivo. *Virology*.

[38] Chang Y. S., Liao C. L., Tsao C. H. (1999). Membrane permeabilization by small hydrophobic nonstructural proteins of Japanese encephalitis virus. *J. Virol*.

[39] McElroy K. L., Tsetsarkin K. A., Vanlandingham D. L., Higgs S. (2006). Manipulation of the yellow fever virus non-structural genes 2A and 4B and the 3′non-coding region to evaluate genetic determinants of viral dissemination from the Aedes Aegypti midgut. *The American Journal of Tropical Medicine and Hygiene*.

[40] Xie X., Gayen S., Kang C., Yuan Z., Shi P.-Y. (2013). Membrane topology and function of dengue virus NS2A protein. *Journal of Virology*.

[41] Liu W. J., Wang X. J., Clark D. C., Lobigs M., Hall R. A., Khromykh A. A. (2006). A single amino acid substitution in the West Nile virus nonstructural protein NS2A disables its ability to inhibit alpha/beta interferon induction and attenuates virus virulence in mice. *Journal of Virology*.

[42] Liu W. J., Wang X. J., Mokhonov V. V., Shi P.-Y., Randall R., Khromykh A. A. (2005). Inhibition of interferon signaling by the New York 99 strain and Kunjin subtype of West Nile virus involves blockage of STAT1 and STAT2 activation by nonstructural proteins. *Journal of Virology*.

[43] Falgout B., Pethel M., Zhang Y.-M., Lai C.-J. (1991). Both nonstructural proteins NS2B and NS3 are required for the proteolytic processing of dengue virus nonstructural proteins. *Journal of Virology*.

[44] Aguirre S., Maestre A. M., Pagni S. (2012). DENV Inhibits Type I IFN Production in Infected Cells by Cleaving Human STING. *PLoS Pathogens*.

[45] Aguilera-Pesantes D., Méndez M. A. (2016). Structure and sequence based functional annotation of Zika virus NS2b protein: Computational insights. *Biochemical and Biophysical Research Communications*.

[46] Zmurko J., Neyts J., Dallmeier K. (2015). Flaviviral NS4b, chameleon and jack-in-the-box roles in viral replication and pathogenesis, and a molecular target for antiviral intervention. *Reviews in Medical Virology*.

[47] Lu G., Gong P. (2013). Crystal Structure of the Full-Length Japanese Encephalitis Virus NS5 Reveals a Conserved Methyltransferase-Polymerase Interface. *PLoS Pathogens*.

[48] Li X.-D., Shan C., Deng C.-L. (2014). The Interface between Methyltransferase and Polymerase of NS5 Is Essential for Flavivirus Replication. *PLOS Neglected Tropical Diseases*.

[49] Klema V. J., Ye M., Hindupur A. (2016). Dengue Virus Nonstructural Protein 5 (NS5) Assembles into a Dimer with a Unique Methyltransferase and Polymerase Interface. *PLoS Pathogens*.

[50] Zhao B., Yi G., Du F. (2017). Structure and function of the Zika virus full-length NS5 protein. *Nature Communications*.

[51] Chang D. C., Hoang L. T., Mohamed Naim A. N. (2016). Evasion of early innate immune response by 2′-O-methylation of dengue genomic RNA. *Virology*.

[52] Davidson A. D. (2009). Chapter 2 New Insights into Flavivirus Nonstructural Protein 5. *Advances in Virus Research*.

[53] Xu X., Vaughan K., Weiskopf D. (2016). Identifying candidate targets of immune responses in Zika Virus based on homology to Epitopes in other Flavivirus Species. *PLoS Currents*.

[54] Shi Y., Gao G. F. (2017). Structural Biology of the Zika Virus. *Trends in Biochemical Sciences*.

[55] Coelho E. D., Arrais J. P., Matos S. (2014). Computational prediction of the human-microbial oral interactome. *BMC Systems Biology*.

[56] Tang H., Hammack C., Ogden S. C. (2016). Zika virus infects human cortical neural progenitors and attenuates their growth. *Cell Stem Cell*.

[57] Arrais J. P., Rosa N., Melo J. (2013). OralCard: A bioinformatic tool for the study of oral proteome. *Archives of Oral Biolog*.

[58] Rosa N., Correia M. J., Arrais J. P. (2012). From the salivary proteome to the OralOme: Comprehensive molecular oral biology. *Archives of Oral Biolog*.

[59] Shannon P., Markiel A., Ozier O. (2003). Cytoscape: a software Environment for integrated models of biomolecular interaction networks. *Genome Research*.

[60] Kanehisa M., Sato Y., Kawashima M., Furumichi M., Tanabe M. (2016). KEGG as a reference resource for gene and protein annotation. *Nucleic Acids Research*.

[61] Kanehisa M., Goto S. (2000). KEGG: kyoto encyclopedia of genes and genomes. *Nucleic Acids Research*.

[62] Amara A., Mercer J. (2015). Viral apoptotic mimicry. *Nature Reviews Microbiology*.

[63] Guirimand T., Delmotte S., Navratil V. (2015). VirHostNet 2.0: Surfing on the web of virus/host molecular interactions data. *Nucleic Acids Research*.

[64] Hulo C., De Castro E., Masson P. (2011). ViralZone: A knowledge resource to understand virus diversity. *Nucleic Acids Research*.

[65] UniProt Consortium (2017). Uniprot: the universal protein knowledgebase. *Nucleic Acids Research*.

[66] Sievers F., Wilm A., Dineen D. (2011). Fast, scalable generation of high-quality protein multiple sequence alignments using Clustal Omega. *Molecular Systems Biology*.

[67] Yoon K., Song G., Qian X. (2017). Zika-Virus-Encoded NS2A Disrupts Mammalian Cortical Neurogenesis by Degrading Adherens Junction Proteins. *Cell Stem Cell*.

[68] Szklarczyk D., Franceschini A., Wyder S. (2015). STRING v10: protein-protein interaction networks, integrated over the tree of life. *Nucleic Acids Research*.

[69] Bhattacharyya S., Zagórska A., Lew E. D. (2013). Enveloped viruses disable innate immune responses in dendritic cells by direct activation of TAM receptors. *Cell Host & Microbe*.

[70] Valadão A. L. C., Aguiar R. S., de Arruda L. B. (2016). Interplay between inflammation and cellular stress triggered by Flaviviridae viruses. *Frontiers in Microbiology*.

[71] Bayer A., Lennemann N. J., Ouyang Y. (2016). Type III Interferons Produced by Human Placental Trophoblasts Confer Protection against Zika Virus Infection. *Cell Host & Microbe*.

[72] de Noronha L., Zanluca C., Azevedo M. L. V., Luz K. G., dos Santos C. N. D. (2016). Zika virus damages the human placental barrier and presents marked fetal neurotropism. *Memórias do Instituto Oswaldo Cruz*.

[73] Tabata T., Petitt M., Puerta-Guardo H. (2016). Zika Virus Targets Different Primary Human Placental Cells, Suggesting Two Routes for Vertical Transmission. *Cell Host & Microbe*.

[74] Miner J. J., Diamond M. S. (2016). Understanding how zika virus enters and infects neural target cells. *Cell Stem Cell*.

[75] Savidis G., McDougall W. M., Meraner P. (2016). Identification of Zika Virus and Dengue Virus Dependency Factors using Functional Genomics. *Cell Reports*.

[76] Nowakowski T. J., Pollen A. A., Di Lullo E., Sandoval-Espinosa C., Bershteyn M., Kriegstein A. R. (2016). Expression analysis highlights AXL as a candidate zika virus entry receptor in neural stem cells. *Cell Stem Cell*.

[77] Gervásio O. L., Phillips W. D., Cole L., Allen D. G. (2011). Caveolae respond to cell stretch and contribute to stretch-induced signaling. *Journal of Cell Science*.

[78] Fibriansah G., Ibarra K. D., Ng T.-S. (2015). Cryo-EM structure of an antibody that neutralizes dengue virus type 2 by locking E protein dimers. *Science*.

[79] Cherrier M. V., Kaufmann B., Nybakken G. E. (2009). Structural basis for the preferential recognition of immature flaviviruses by a fusion-loop antibody. *EMBO Journal*.

[80] Richard A. S., Shim B.-S., Kwon Y.-C. (2017). AXL-dependent infection of human fetal endothelial cells distinguishes Zika virus from other pathogenic flaviviruses. *Proceedings of the National Acadamy of Sciences of the United States of America*.

[81] Dejnirattisai W., Webb A. I., Chan V. (2011). Lectin switching during dengue virus infection. *The Journal of Infectious Diseases*.

[82] Schneider W. M., Chevillotte M. D., Rice C. M. (2014). Interferon-stimulated genes: a complex web of host defenses. *Annual Review of Immunology*.

[83] Thiemmeca S., Tamdet C., Punyadee N. (2016). Secreted NS1 protects dengue virus from mannose-binding lectin-mediated neutralization. *The Journal of Immunology*.

[84] Suthar M. S., Aguirre S., Fernandez-Sesma A. (2013). Innate Immune Sensing of Flaviviruses. *PLoS Pathogens*.

[85] Matsumiya T., Stafforini D. M. (2010). Function and Regulation of Retinoic Acid-Inducible Gene-I. *Critical Reviews™ in Immunology*.

[86] Kato H., Takeuchi O., Sato S. (2006). Differential roles of MDA5 and RIG-I helicases in the recognition of RNA viruses. *Nature*.

[87] Gack M. U. (2014). Mechanisms of RIG-I-Like receptor activation and manipulation by viral pathogens. *Journal of Virology*.

[88] Nasirudeen A. M. A., Wong H. H., Thien P., Xu S., Lam K.-P., Liu D. X. (2011). RIG-I, MDA5 and TLR3 synergistically play an important role in restriction of dengue virus infection. *PLOS Neglected Tropical Diseases*.

[89] Quicke K. M., Bowen J. R., Johnson E. L. (2016). Zika Virus Infects Human Placental Macrophages. *Cell Host & Microbe*.

[90] Best S. M. (2017). The many faces of the flavivirus NS5 protein in antagonism of type I interferon signaling. *Journal of Virology*.

[91] Ashour J., Laurent-Rolle M., Shi P.-Y., García-Sastre A. (2009). NS5 of dengue virus mediates STAT2 binding and degradation. *Journal of Virology*.

[92] Grant A., Ponia S. S., Tripathi S. (2016). Zika Virus Targets Human STAT2 to Inhibit Type i Interferon Signaling. *Cell Host & Microbe*.

[93] Dar H. A., Zaheer T., Paracha R. Z., Ali A. (2017). Structural analysis and insight into Zika virus NS5 mediated interferon inhibition. *Infection, Genetics and Evolution*.

[94] Versteeg G. A., Rajsbaum R., Sánchez-Aparicio M. T. (2013). The E3-ligase TRIM family of proteins regulates signaling pathways triggered by innate immune pattern-recognition receptors. *Immunity*.

